# Absence of a home-field advantage within a short-rotation arable cropping system

**DOI:** 10.1007/s11104-022-05419-z

**Published:** 2022-04-26

**Authors:** Marijke Struijk, Andrew P. Whitmore, Simon Mortimer, Xin Shu, Tom Sizmur

**Affiliations:** 1 Department of Geography and Environmental Science, University of Reading, Reading, UK; 2 Department of Sustainable Agriculture Sciences, Rothamsted Research, Harpenden, UK; 3 School of Agriculture, Policy and Development, University of Reading, Reading, UK

**Keywords:** Home-field advantage, Decomposition, Crop residues, Legacy effect, Arable cropping system

## Abstract

**Aims:**

The home-field advantage (HFA) hypothesis predicts faster decomposition of plant residues in *home* soil compared to soils with different plants (*away*), and has been demonstrated in forest and grassland ecosystems. It remains unclear if this legacy effect applies to crop residue decomposition in arable crop rotations. Such knowledge could improve our understanding of decomposition dynamics in arable soils and may allow optimisation of crop residue amendments in arable systems by cleverly combining crop-residue rotations with crop rotations to increase the amount of residue-derived C persisting in soil.

**Methods:**

We tested the HFA hypothesis in a reciprocal transplant experiment with mesh bags containing wheat and oilseed rape residues in soils at three stages of a short-rotation cropping system. Subsets of mesh bags were retrieved monthly for six months to determine residue decomposition rates, concomitantly measuring soil available N, microbial community structure (phospholipid fatty acid analysis), and microbial activity (Tea Bag Index protocol) to assess how plants may influence litter decomposition rates via alterations to soil biochemical properties and microbial communities.

**Results:**

The residues decomposed at similar rates at all rotational stages. Thorough data investigation using several statistical approaches revealed no HFA within the crop rotation. Soil microbial community structures were similar at all rotational stages.

**Conclusions:**

We attribute the absence of an HFA to the shortness of the rotation and soil disturbance involved in intensive agricultural practices. It is therefore unlikely that appreciable benefits could be obtained in short conventionally managed arable rotations by introducing a crop-residue rotation.

**Supplementary Information:**

The online version contains supplementary material available at 10.1007/s11104-022-05419-z.

## Introduction

Soil organic matter (SOM) has been identified as a major factor for improving the ability of soils to sustain crops and provide ecosystem services like climate regulation (Kibblewhite et al. 2008). However, croplands have added an estimated 98.4 Pg of C to the atmosphere between 1850 and 2015 (Houghton and Nassikas 2017). Crop residues – straw, stalks, leaves, etc. – comprise the majority of plant materials harvested worldwide, with an estimated annual production ranging from 3.4 Pg to 3.8 Pg (Lal 1997; Smil 1999), and thus represent a considerable opportunity to increase SOM. Wheat residues in particular, have a higher carbon content (around 46%) than other soil organic amendments (Sizmur et al. 2017). With an annual production estimated as 0.85 Pg yr^−1^ to 0.96 Pg yr^−1^, wheat residues constitute roughly a quarter of the world’s crop residues (Lal 1997; Smil 1999).

Current thinking recognises the important role of microbial metabolites and necromass in soil C accumulation, as opposed to unmineralised residues (Ma et al. 2018; Liang et al. 2019). Just a small increase in the proportion of wheat straw, and other crop residues, that is converted into SOM, mainly via microbial assimilation, could have a large impact on the global atmospheric C loading. If just 1% of the world’s crop residues (3750 Tg yr-1 in the mid 1990s; Smil 1999) could be turned into SOC on arable land (ca. 1417 Mha in 2014; FAO 2014), that would correspond to a 2.6 g C m^−2^ yr^−1^ short-term increase in SOC in arable soils, leading to an estimated 0.3 g C m^−2^ yr^−1^ long-term increase, assuming about 12.5% of the SOC remains stable after 10 years, as was determined for ryegrass by Jenkinson (1977). In comparison, no-till agriculture has an estimated potential of 0.03 g C m^−2^ yr^−1^ increase in SOC (Powlson et al. 2014).

Apart from being used as biofuels, and animal feed and fodder, crop residues represent a major on-farm resource that could be applied as a soil amendment. Transformation of unmineralized residues into SOM, via microbial decomposition and direct litter consumption by other saprotrophic organisms, “feeds” the soil food web and soil organisms of higher trophic levels, in turn, feed on these microorganisms and saprotrophs. The members of the soil food web are involved in soil aggregation, nutrient cycling, and improving the conditions for primary production. Unfortunately, straw incorporation is often inefficient in terms of SOM formation, as demonstrated by studies that compare straw to other soil amendments for their C accumulation potential (Powlson et al. 2012), or studies that compare CO_2_ savings by soil organic carbon (SOC) formation from straw amendments to biofuel use to displace electricity generated from fossil fuels (Powlson et al. 2008). This inefficiency is likely because straw has a low carbon use efficiency, meaning that a high proportion of the carbon within the straw is respired as CO_2_ rather than incorporated into microbial biomass where it could become necromass and stable soil organic matter (Dannehl et al. 2017). However, these results are based on experiments in which crops were grown and their residues applied to soils directly after that crop’s harvest. Perhaps a different application strategy could be devised, based on a better understanding of decomposition processes in arable cropping soils, to increase the amount of straw-derived C persisting in the soil and to decrease the amount of C that is lost.

According to the legacy effect described by the home-field advantage (HFA) hypothesis, plant residues are predicted to decompose faster in the soil in which they were grown (*home*) compared to a soil where different plants were grown (*away*) (Gholz et al. 2000). This has previously been observed within forest (Ayres et al. 2009) and grassland (Rashid et al. 2013) ecosystems, as well as between cropland, grassland and forest biomes (Di Lonardo et al. 2018). However, little is known about applicability of the HFA hypothesis to rotational arable cropping systems (but see Barel et al. 2019). The HFA hypothesis is attributed to adaptation and optimisation of the soil microbial community to *home* plants’ residues (Austin et al. 2014; Ayres et al. 2009), which is based primarily on the observations that the soil microbial community is not entirely functionally redundant (Strickland et al. 2009a, b) and that different soil microorganisms have different metabolic capacities (Keiser et al. 2014; Wickings et al. 2012). However, the mechanisms involved in the formation of a *home* microbial community remain poorly understood (Austin et al. 2014) and therefore the validity of the HFA between different years of typical arable cropping systems, which are different in nature to forests and grasslands in terms of the time that the soil is exposed to a particular crop, is difficult to predict.

In forest and grassland conditions, the litter decomposition rate could theoretically be manipulated by selecting *home* or *away* litter. If *away* litter is applied, effectively realising an “away-field disadvantage,” the soil microbial community is not adapted to being able to easily decompose the *foreign* litter. At this lower decomposition rate, the “unfamiliar” organic substrates are not easily decomposable, temporarily realising resource-poor conditions in which K-strategists, which tend to exhibit a higher overall carbon use efficiency than *r*-strategists, are favoured (Fierer et al. 2007; Kallenbach et al. 2019). Therefore, realising an “away-field disadvantage” might increase the net accumulation of SOM from crop residue amendments compared to application of litters in *home* soil. However, this objective could only be accomplished in an arable cropping system if an HFA is found in this environment. Therefore, determination of the applicability of the HFA hypothesis to arable cropping systems could inform strategies for optimal crop residue applications in arable cropping systems.

In this experiment we tested the HFA hypothesis within an intensively managed arable cropping system of continuous wheat with an oilseed-rape (OSR) break crop every four years. Each stage in this crop rotation was represented in the experimental plots every year by using a space-for-time substitution. Wheat and OSR residues were buried in 1^st^ wheat, 2^nd^ wheat and OSR plots, and their mass loss measured over time over a period of six months (during the growing season). Soil available N and the microbial community structure (by phospholipid fatty acid analysis) were assessed as explanatory variables. In line with the HFA hypothesis, we hypothesised that (1) wheat straw incorporated in a soil after a wheat crop would decompose faster than wheat straw incorporated after an OSR crop. Likewise, OSR straw incorporated after an OSR crop would decompose faster than OSR straw incorporated after a wheat crop; and that (2) the composition of the belowground soil microbial community would be altered by the crops grown aboveground.

## Methods

### Study site

**Fig. 1:**
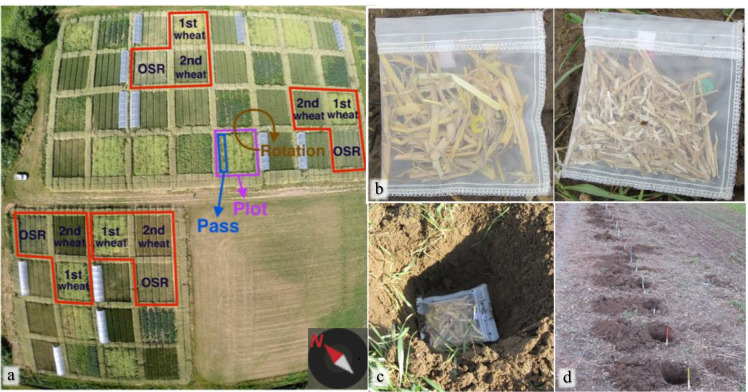
**a** Aerial photograph of the field site, indicating the plots (12 × 10 m^2^) of the intensive arable rotation included in this study. Rotational stage of each plot in the experiment indicated in black text. Image taken by Richard Casebow. **b** Mesh bags containing wheat (left) and OSR (right) straw. **c** Mesh bag buried at 15-cm depth (bottom left). **d** A pass in an OSR plot with 15-cm deep holes for mesh bags, locations marked with peg

This experiment was carried out in 2016 on an established field experiment at the University of Reading’s Sonning Farm, UK (51.481152, -0.902188). The field experiment was established in 2013, succeeding many years of grass ley followed by one season of winter barley and one season of winter wheat. The soil at Sonning Farm has a free-draining sandy/silty loam texture containing around 5.6% clay, 50.7% silt and 43.7% sand (Degani et al. 2019). The HFA was tested in a simple crop rotation (Fig. 1a) representative of an intensive agricultural system in the UK which was part of a larger experiment described fully elsewhere (Degani et al. 2019). The rotation comprised Winter Wheat (*Triticum* sp., var. Solstice) and OSR (*Brassica napus* sp., var. Tamarin). Three stages of the crop rotation (treatments) were selected: 1^st^ wheat (i.e. the first wheat crop after OSR), 2^nd^ wheat (i.e. the second wheat crop after OSR) and OSR break crop (indicated in bold in Table 1). Each rotation was replicated four times and the experiment blocked, with one replicate rotation (comprising four plots, each at a different stage) in each block. We employed treatment labels as [previous crop]-[current crop], as follows: OSR-WW for 1^st^ wheat, WW-WW for 2^nd^ wheat, and WW-OSR for OSR (Table 1). For clarity, this treatment description was used because we hypothesised that an HFA effect would be based on a legacy effect of the previous year’s crop. The 3^rd^ wheat crop rotation stage was excluded from this study because it was used for another experiment.

**Table 1:** Cropping history per rotational stage included in this study. Rotation stages and treatment labels used in this study are indicated in bold

Up to 2011	Grass ley	Grass ley	Grass ley
2011–2012	Winter barley	Winter barley	Winter barley
2012–2013	Winter wheat	Winter wheat	Winter wheat
2013–2014	3^rd^ wheat	OSR	2^nd^ wheat
2014–2015	OSR	1^st^ wheat	3^rd^ wheat
**2015–2016**	**1**^**st**^** wheat**	**2**^**nd**^** wheat**	**OSR**
**Treatment label**	**OSR-WW**	**WW-WW**	**WW-OSR**

Abbreviations: *OSR* is oilseed rape, *WW* is winter wheat

For the established field experiment in which our experiment was performed (see Degani et al. 2019), the researchers opted for a nitrogen fertilisation rate at 50% of the recommended rate (according to RB209 Fertiliser Manual; Defra, UK), i.e. 50 kg N + 50 kg SO_3_ per ha, applied as ammonium nitrate (34.5% N) and ammonium sulphate nitrate (26% N, 37% SO_3_). This rate was selected because the researchers wanted to find out if diversity in the crop rotation can improve nutrient use efficiency. Wheat plots were fertilised on 8 April and OSR plots were fertilised on 20 April (see Online Resource section S1 for a schedule of the experiment).

### Mesh bag preparation, burial and retrieval

Wheat and OSR residues were collected from harvests from the 2015 growing seasons. To obtain a more homogenous material, nodes and ears were removed from the wheat residues and big stalks were removed from OSR residues. A series of wheat- and OSR straw samples (residue types) were buried, as specified in the following paragraphs, in plots cropped with 1^st^ wheat, 2^nd^ wheat and OSR (treatments; OSR-WW, WW-WW and WW-OSR, respectively). Sub-samples of the crop residues were dried at 60 ºC, milled to a fine powder, and analysed for total C and N with a Flash 2000, Thermo Fisher Scientific, Cambridge, UK. We obtained a 109% recovery of both C and N for in-house reference material traceable to certified reference materials. The total concentrations of micronutrients were determined by ICP-OES (inductively coupled plasma optical emission spectroscopy; Perkin Elmer Optima 7300 Dual View) analysis of 0.5 g residues samples digested in 8 ml of nitric acid using MARS 6 microwave digestion system (Table 2). We obtained recovery rates of 99% (P), 94% (K), 102% (Mg), 114% (Fe), 104% (Zn), 96% (Ca), and 92% (Mn) from analysis of an in-house hay reference material traceable to certified reference NCSDC 73,349).

**Table 2:** Residue characterisation (SEM included in parentheses; n = 3)

Nutrient	Wheat straw	OSR straw	Rooibos tea^1^	Green tea^1^
C (g/kg)	454.1 (1.60)	463.0 (1.16)	505.1 (1.7)	490.6 (0.6)
N (g/kg)	6.6 (0.05)	5.0 (0.03)	11.9 (0.3)	40.2 (0.3)
C:N	68.5 (0.41)	93.3 (0.6)	42.9 (1.06)	12.2 (0.07)
P (mg/kg)	1433 (7.0)	694 (10.1)		
K (mg/kg)	14,341 (59.3)	2965 (34.5)		
N:P	0.0046	0.0072		
Mg (mg/kg)	649 (1.9)	196 (1.1)		
Fe (mg/kg)	653 (11.5)	132 (3.0)		
Zn (mg/kg)	13 (0.3)	5 (0.3)		
Ca (mg/kg)	6578 (22.6)	17,077 (216.3)		
Mn (mg/kg)	65 (0.5)	10 (0.1)		

^1^Data taken from (Keuskamp et al. 2013)

Mesh bags with the residues were prepared on 6 and 7 February 2016, and buried at 15 cm depth, to reflect the depth it would be buried if the crop residues were ploughed in, on 10 (i.e. day 1 of experiment) and 12 February 2016 (Online Resource section S1). A total of 144 mesh bags containing OSR residues and 144 mesh bags containing wheat residues were buried. Twelve mesh bags containing wheat residues and twelve containing OSR residues were buried in each of the twelve plots (three treatments replicated four times). Every four weeks, two wheat mesh bags and two OSR mesh bags were retrieved, resulting in six retrievals over 24 weeks in total. Along with a numbered colour-coded identification tag, 5 g (± 5%) of residues – OSR or wheat straw (internodes only) – were inserted into each mesh bag (Schwegmann Filtrations-Technik, polyamide monofilament, 500 µm mesh size, 12 cm × 12 cm; Fig. 1b). A 500 µm mesh was chosen to allow access to microorganisms and enable microbial decomposition, but exclude most mesofauna and avoid loss of small undecomposed residue fragments (Bradford et al. 2002; Castro-Huerta et al. 2015). Mesh bags were sealed with 100% polyester sewing thread. Mesh bag “blanks” were also performed by burying and directly thereafter retrieving 10 bags of each residue, to account for mass loss in the process of transporting, burying and retrieving mesh bags, without decomposition of the residues.

Mesh bags were retrieved with a spade 26, 54, 82, 110, 138, and 166 days after burial (Online Resource section S1), dried at 50 ºC, and subsequently cut open to sort the residues. This involved removing soil and roots that had entered the mesh bag as much as possible and placing the residues in a pre-dried and pre-weighed paper bag for final drying of residues at 60 ºC overnight. Dried bags were cooled in a desiccator and re-weighed. Drying, desiccating and re-weighing were repeated and the average weight taken. Finally, because some soil had entered the mesh bags and was stuck to the residues, each residue sample was ashed overnight in a crucible at 550 ºC to account for the mineral content and determine the ash-free dry mass.

To assess the baseline decomposition rate in each plot, and by extension the soil microbial activity, Lipton Green and Rooibos teabags were buried on 22 May 2016 (102 days after mesh bag burial) and retrieved on 8 August 2016 (180 days after mesh bag burial), following the Tea Bag Index (TBI) protocol (Keuskamp et al. 2013). During this period, all labile substrates from Green tea are expected to be decomposed, but the fraction of the labile substrates that are actually decomposed depends on environmental factors that may lead to stabilisation (recalcitrance) of some of the labile compounds. This allows for calculation of the stabilisation factor *S*_*TBI*_ by comparing the decomposed fraction of Green tea measured in the field (*a*_*g*_) to the expected labile fraction of Green tea from its chemical composition (hydrolysable fraction *H*_*g*_ determined by Keuskamp et al. (2013)):

$${S}_{TBI}=1-\frac{{a}_{g}}{{H}_{g}}$$

Rooibos tea is less decomposable and labile fractions are still expected to be decomposing when the teabags are retrieved, allowing for estimation of the decomposition rate constant *k*_*TBI*_. Greater decomposition implies less stabilisation, and vice versa (for further details, see Keuskamp et al. 2013).

Tea has never been grown at the site, so it was deemed to be a foreign substrate to the soil microbial community in all the plots, such that an HFA effect would not apply. Thus, the TBI serves as a general assessment of the inherent activity of the soil microbial community.

### Soil sampling

Soil sampling was performed to 20-cm depth in a zig-zag fashion using a 30-mm diameter gouge auger. Soils were sieved to 4 mm immediately after sampling and stored at 4 ºC. Initial soil samples were taken on 12 and 15 February 2016 to assess baseline conditions. Subsequent soil samples were taken concomitant with each mesh bag retrieval and analysed for soil available N to determine soil conditions in each plot over time.

Available N (i.e. sum of NO_3_^−^ and NH_4_^+^) was extracted from field moist soil with a mass equivalent to 40 g of dry-soil by shaking for 30 min in 200 ml 1 M KCl (99.5% purity). Extracts were filtered through Whatman no. 2 filters and analysed colourimetrically for nitrate and ammonia on a Skalar San +  + continuous flow analyser. Available N was calculated per gram of dry soil extracted and taken as the sum of the NO_3_^−^ and NH_4_^+^ measured in the extract.

### PLFA analysis

Additional 20-cm depth soil samples were taken with a 30-mm diameter gauge auger in a zig-zag fashion alongside the mesh bag locations from every plot at the beginning (4 March 2016) and end (5 July 2016) of the mesh bag burial period. Subsamples of these soil samples were immediately frozen, and subsequently freeze-dried to assess microbial community structure using phospholipid fatty acid (PLFA) profiles, following Sizmur et al. (2011). This method exploits the fact that fungi, gram-negative, gram-positive, mycorrhizal fungi and actinomycetes each exhibit PLFAs with different structural compositions. Soils were extracted using Bligh and Dyer solvent (Bligh and Dyer 1959) according to Frostegård and Bååth (1996), extracted phospholipids were derivatised according to Dowling et al. (1986) and analysed as fatty acid methyl esters by gas chromatography (Agilent 6890 N, flame ionisation detector and a 30 m × 0.25 mm capillary column with a 0.25 μm film of 5% diphenyl, 95% dimethyl siloxane) according to Frostegård et al. (1991). The internal standards used were methyl tetradecanoate (C14:0; Sigma-Aldrich) and methyl nonadecanoate (C19:0; Sigma-Aldrich; 96.0% purity).

PLFA chromatograms were integrated and peaks identified according to the retention time and peak area based on Bacterial Acid Methyl Ester (BAME) and Fatty Acid Methyl Ester (FAME) chromatograms provided by the supplier of the standards (Supelco, Supelco UK, Poole, UK), as well as soil-sample chromatograms with peaks previously identified using GC–MS at Cranfield University. A broad selection of peaks between C14 and C20 were included in further data analyses, following the classification in Table 3. Concentrations of fatty acid methyl esters were calculated based on FAME calibration standards, and data were adjusted to blanks (noise/contamination) and the internal standard C19:0. We hereafter refer to fatty acid methyl ester biomass as Total PLFAs. Although we recognise the shortcomings of this approach (see e.g. Strickland and Rousk 2010), the fungal:bacterial (F:B) ratio was calculated based on the classification of PLFAs specified above and using the amount of PLFAs calculated from the FAME calibration. Previous studies have attempted to determine a conversion factor for fungal biomass from PLFA analysis (e.g. Bååth and Anderson 2003; Frostegård et al. 1991; Klamer and Bååth 2004) but, due to the lack of agreement, we have not applied any conversion factor and calculated F:B simply as the quotient of the amount of fungal and bacterial fatty acids as determined by the method specified above. This will provide some indication of the differences between treatments and differences over time in these microbial groups.

**Table 3:** Classification of PLFA fatty acids included in analyses

Bacterial			Fungal	Protist	Other
Gram +	Gram –	Other			
i14:0	16:1ω7t	15:0	18:2ω6	20:4ω6	14:1ω9c
i15:0	17:0cy	17:0	18:1ω9c		i16:1
a15:0	19:0cy				16:1ω11t
i16:0					16:1ω7c
16:1ω5					16:0
i17:0					17:0brα
					17:0brβ
Actinomycetes:					17:1ω8c
10me-17:0					17:1ω7
10me-18:0					12me-17:0
					18:3(5,10,12)
					18:1ω9t
					18:1ω13
					18:1ω10or11
					18:0
					19:1ω6
					19:1ω8
					19:0
					20:5ω3
					20:1ω9
					20:0

Classification according to Bååth and Anderson 2003; Bardgett et al. 1999; Frostegård et al. 1993; Frostegård and Bååth 1996; Kaur et al. 2005; and Kominoski et al. 2009

### Data analyses

Statistical analyses were performed in R 3.5.1 (R Foundation for Statistical Computing) using RStudio 1.1.456 (RStudio, Inc.), GenStat 18.2.0.18409 (VSN International, 2016; used for repeated measures ANOVAs only), and SAS 9.4 (Intel Corporation, 2016; used to fit the HFA model proposed by Keiser et al. (2014) only).

Initial and final weights of residues (adjusted for ash content) were fit to a first-order rate of decay function: *M*_*t*_ = *M*_*0*_*e*^*−kt*^ (M_t_ – residue mass at time t, M_0_ – initial residue mass, k – rate of decay per day). The decomposition rate constant (*k*) was derived per residue per plot and the mean *k* was calculated for each residue per treatment to assess the presence of an HFA effect for each residue type.

The main test to determine if the HFA hypothesis applied was a two-way analysis of variance (ANOVA) of *k*, using the factors soil treatment and residue type, where a significant interaction between treatment and residue would indicate presence of an HFA effect. Blocking was accounted for as a random factor. Subsequently, multiple one- and two-way ANOVAs with and without a range of covariates (e.g. TBI, the average of the available N levels) and with and without blocking as a random factor were performed to investigate the presence of an HFA effect for both or either of the residues. The (observed) M_t_/M_0_ over time was analysed by a repeated measures ANOVA, with and without a range of covariates. We also analysed the data according to the model developed by Keiser et al. (2014), which accounts for the ability of the soil microbial community to decompose substrates, the decomposability of the residue, and the HFA effect by defining mass loss = soil ability + litter ability + home interaction (HFA).

The TBI was calculated according to Keuskamp et al. (2013), producing the decomposition rate constant (*k*_*TBI*_) and stabilisation factor (*S*_*TBI*_) of the soil in each plot. In addition, the mass loss of Green and Rooibos tea was calculated separately based on the initial and final tea mass.

Quantified PLFA data were normalised, and analysed by nonmetric multidimensional scaling (NMDS) ordination using the vegan package in R (v2.5–5; Oksanen et al. 2019).

## Results

### Residue decomposition

**Fig. 2:**
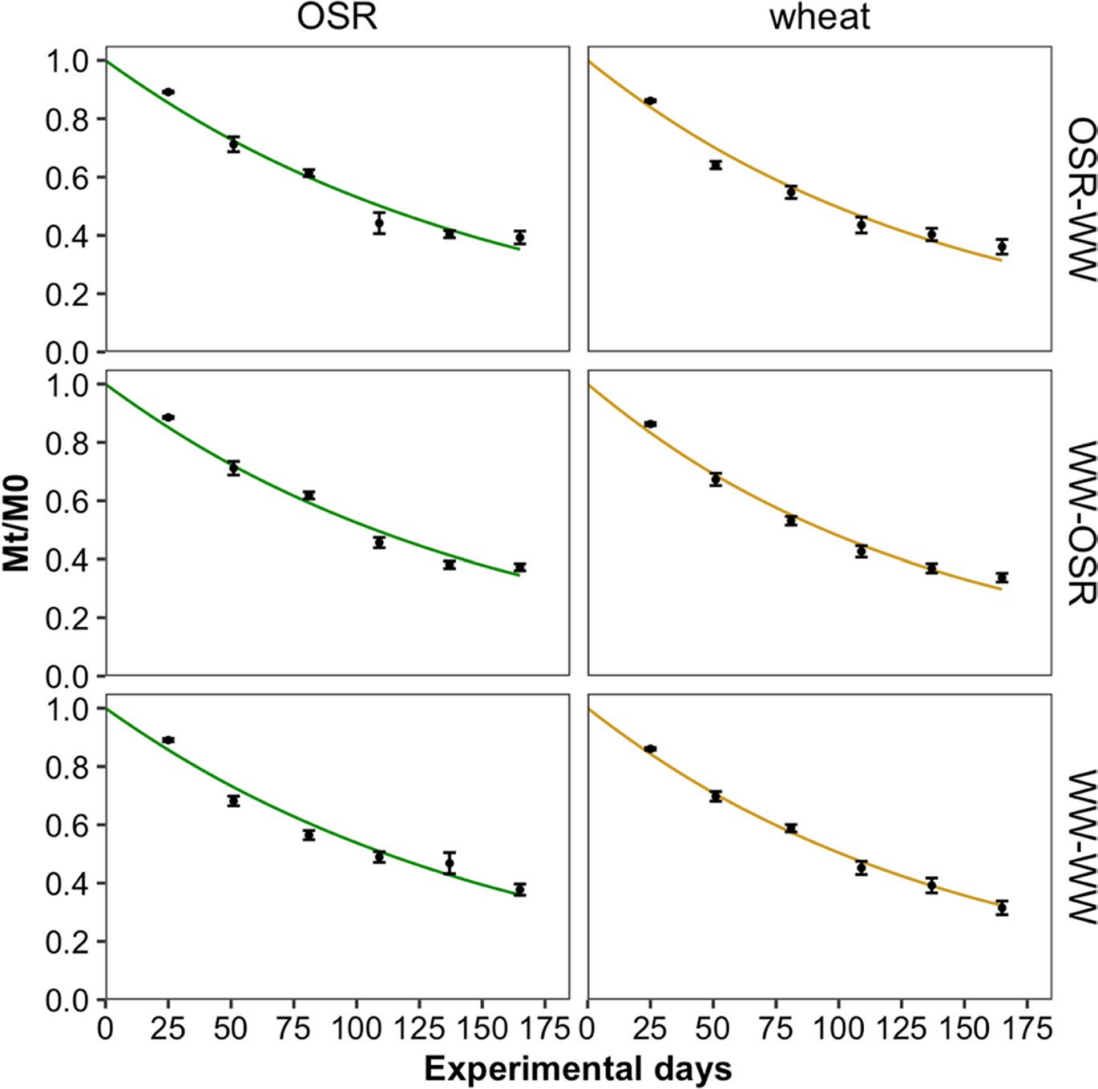
Average remaining residue fraction (M_t_/M_0_) over time with the observed residue fraction remaining (●) fitted to a first order rate of decay model (**—**). Residue types and soil treatments are indicated along the top and right-hand side, respectively. Error bars represent standard error of the mean (n = 8)

**Fig. 3:**
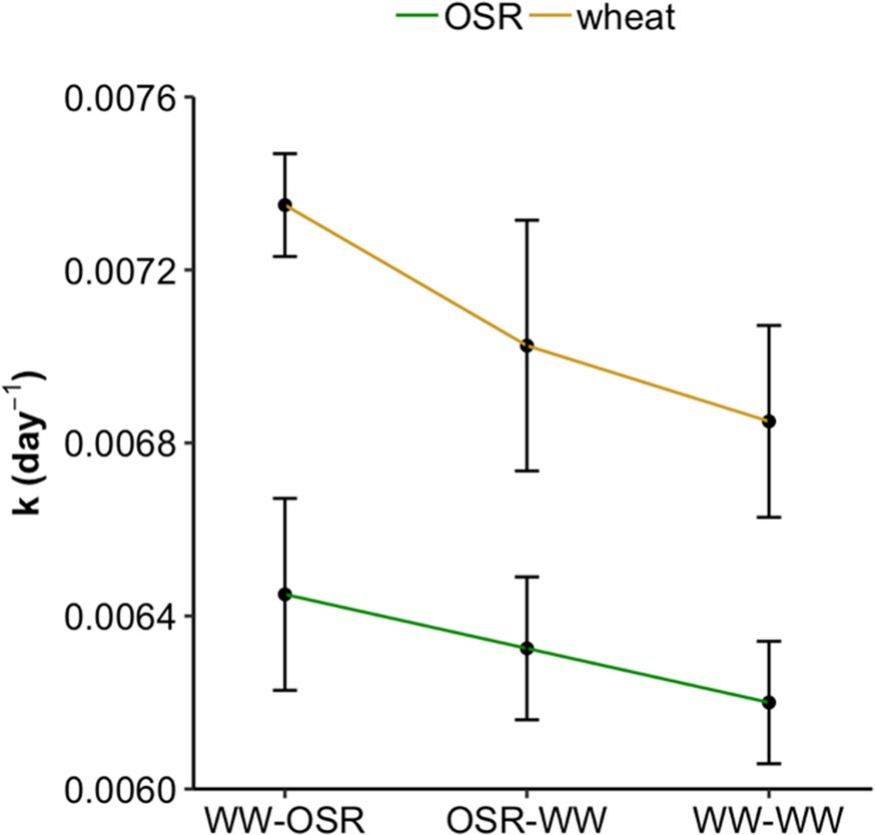
Mean fitted decomposition rate constant (*k*) to assess presence of an HFA effect between treatments per residue. Error bars represent standard error of the mean (n = 4)

Decomposition of wheat and OSR residues buried in the different treatments followed a first-order rate of decay (Fig. 2). About 62% of OSR and 66% of wheat residues decomposed during the experiment. The relative mass loss (M_t_/M_0_) over time did not differ significantly between treatments for each residue (OSR: F_2,6_ = 0.17, p = 0.84; wheat: F_2,6_ = 1.02, p = 0.40; repeated measures ANOVA) and data analysis with available N as a covariate also did not result in the emergence of significant differences between treatments. The decomposition rate constant (*k*) of both residues followed a similar pattern (Fig. 3), decomposing fastest in WW-OSR > OSR-WW > WW-WW (see Table 1 for treatment labels), although there were no significant differences between treatments (F_2,15_ = 1.582, p = 0.238; two-way ANOVA). Decomposition rates of wheat residues were significantly higher than the decomposition rates of OSR residues (F_1,15_ = 18.738, p < 0.001; two-way ANOVA). After thorough investigation of the data by means of ANOVAs with a range of covariates (Online Resource section S2), no HFA effect could be detected, since there was no significant interaction between treatment and residue type. In addition, the relative mass (M_t_/M_0_) of residue remaining at the end of the experiment was analysed according to the model proposed by Keiser et al. (2014), which takes the ability of the soil decomposer community and the decomposability of the residues into account. This analysis revealed no HFA effect either (Online Resource section S5).

### Soil biochemical properties

**Fig. 4:**
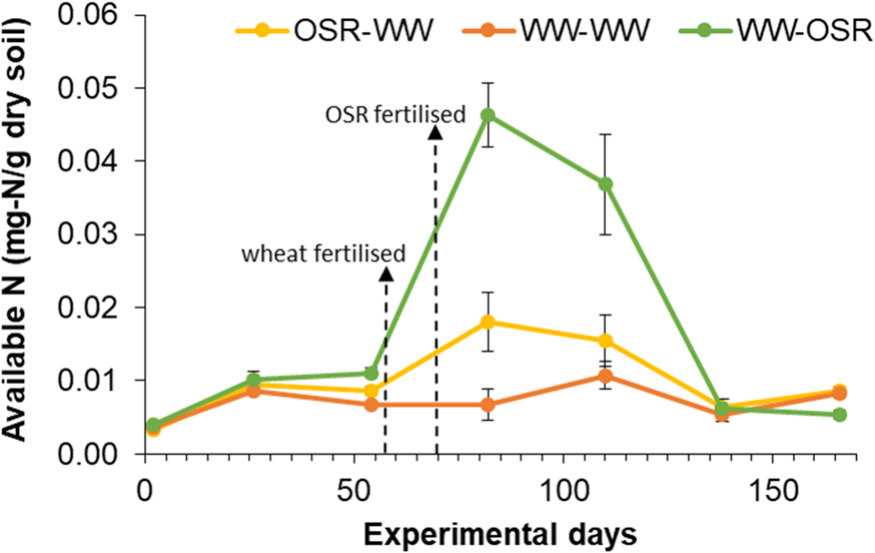
Soil available N per treatment over the course of the experimental period. Error bars represent SEM (n = 4). Timings of fertilisation indicated

Soil available N was monitored during the experimental period concomitant with each mesh bag retrieval instance. Available N levels differed significantly over time (p < 0.001; repeated measures ANOVA) increasing after fertilisation and then decreasing to the original level (Fig. 4). The wheat crops were fertilised earlier than the OSR crops. While this difference may have caused a disproportionately greater available N level in the WW-OSR treatment in month 3, the greater available N levels in the WW-OSR treatment persisted during the subsequent months, suggesting the OSR crops did not grow well (which was observed) and/or greater N retention in the WW-OSR treatment. As a result, we observed significantly different available N levels between treatments (F_2,6_ = 25.02, p < 0.001) and over time (F_5,45_ = 31.07, p < 0.001), and a significant interaction between time and treatment (F_10,45_ = 14.48, p < 0.001; repeated measures ANOVA).

**Fig. 5:**
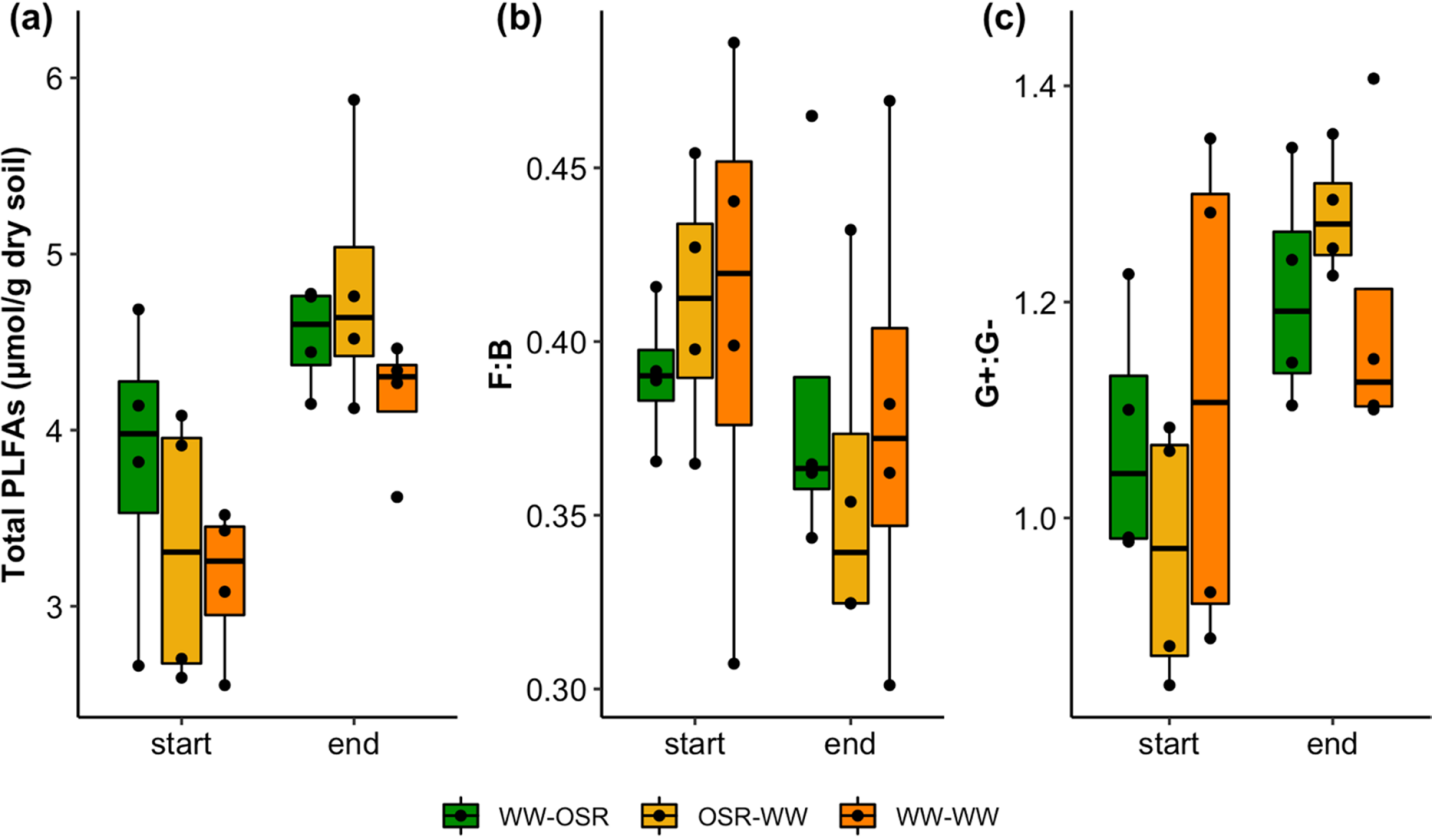
Total PLFAs per treatment at the start (March 2016, 23 days after mesh bag burial) and end (July 2016, 146 days after mesh bag burial) of the mesh bag burial period (**a**); fungal:bacterial (F:B) ratio of fatty acids per treatment at the start (March 2016) and end (July 2016) of the mesh bag burial period (**b**); Gram + :Gram- ratio of fatty acids per treatment at the start (March 2016) and end (July 2016) of the mesh bag burial period (**c**). Lower and upper hinges correspond to the 25th and 75th percentiles; black dots represent individual datapoints (n = 4)

At the start of the growing season (23 days after mesh bag burial), the total amount of PLFAs was greatest in the WW-OSR treatment and lowest in the WW-WW treatment (Fig. 5a). At the end of the mesh bag burial period (23 days after mesh bag burial), the total amount of PLFAs was slightly greater in the OSR-WW treatment compared to WW-OSR, and still lowest in the WW-WW treatment. However, differences in PLFA amounts between treatments at the same time point were not significant (start: F_2,6_ = 0.92, p = 0.45; end: F_2,6_ = 2.14, p = 0.20; one-way ANOVA). In all treatments, the total PLFA amount increased significantly by the end of the experimental period (F_1,18_ = 17.87, p < 0.001; two-way ANOVA), with the greatest increase observed in the OSR-WW treatment.

The fungal:bacterial (F:B) ratio did not change significantly between the start and the end of the experimental period (Fig. 5b) (F_1,18_ = 1.72; p = 0.21; two-way ANOVA). The decrease in F:B over time was lowest in the WW-OSR treatment. Between the treatments, the F:B ratios were quite similar (start: F_2,6_ = 0.202, p = 0.823; end: F_2,6_ = 0.171, p = 0.847; one-way ANOVA), although the WW-WW treatment exhibited the highest F:B ratio at both the start and end of the experimental period. There was a relatively greater increase in bacterial fatty acids during the experimental period compared to fungal fatty acids (Online Resource section S4). The ratio of Gram positive to Gram negative (G + :G–) fatty acids (Fig. 5c) also differed significantly over time (F_1,20_ = 8.68, p = 0.008; two-way ANOVA), but not between treatments (start: F_2,6_ = 0.996, p = 0.423; end: F_2,6_ = 0.663, p = 0.549).

**Fig. 6:**
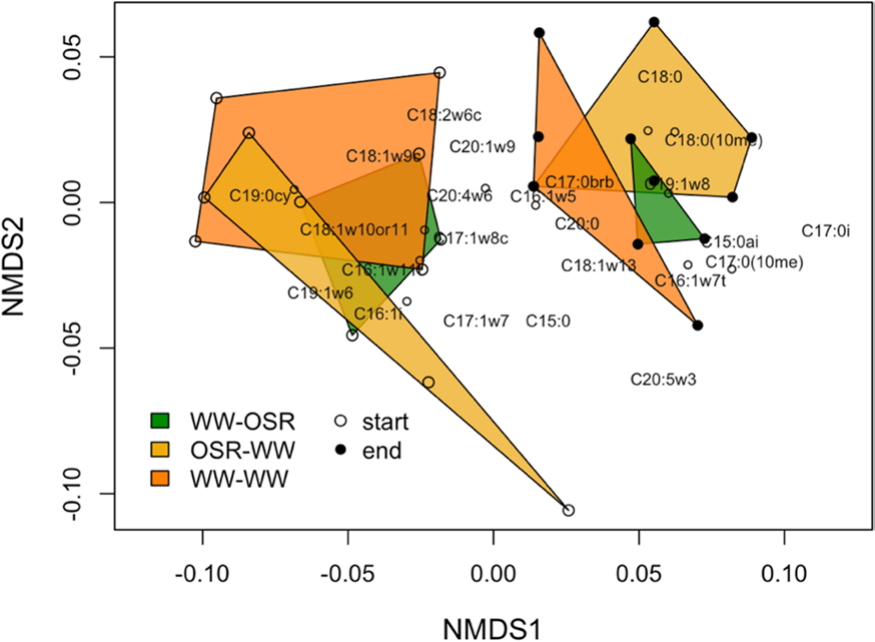
Nonmetric multidimensional scaling (NMDS) ordination of relative abundances of identified fatty acids at the start (23 days after mesh bag burial) and end (146 days after mesh bag burial) of the experimental period (based on Bray–Curtis dissimilarity matrix; stress = 0.12). Each circle represents a PLFA profile of a replicate plot of the treatments. Circles that are closer together exhibit more similar microbial communities. Vectors of individual fatty acids are also plotted with labels

Ordination of the PLFA profiles enabled comparison of the microbial community structure between treatments (Fig. 6). This comparison revealed a clear separation in the microbial communities that were present at the start versus the end of the mesh bag burial period (F_1,22_ = 11.24, p < 0.01; PERMANOVA). At each time point (start and end) the polygons in the ordination plot show considerable overlap, indicating that the microbial communities did not differ notably between the treatments at each time point (F_2,22_ = 0.91, p = 0.50; PERMANOVA).

The amount of some individual fatty acids did differ significantly (p < 0.05) or noticeably (p < 0.1) between treatments: C15:0ai, C16:0i, C16:0, C17:0brβ, C17:1ω7, C18:1ω13, C19:0cy, and C20:0 (a combination of G + and G– bacterial biomarkers) (see Online Resource section S4). The amount of these fatty acids followed a similar trend between treatments; they were highest in WW-OSR > OSR-WW > WW-WW at the start of the mesh bag burial period, and highest in OSR-WW > WW-OSR > WW-WW at the end of the mesh bag burial period. A two-way ANOVA of the effects of time and treatment on these fatty acids combined shows that the WW-OSR treatment and the WW-WW treatment were significantly different from each other (p = 0.037, post-hoc Tukey HSD).

### Tea Bag Index

**Fig. 7:**
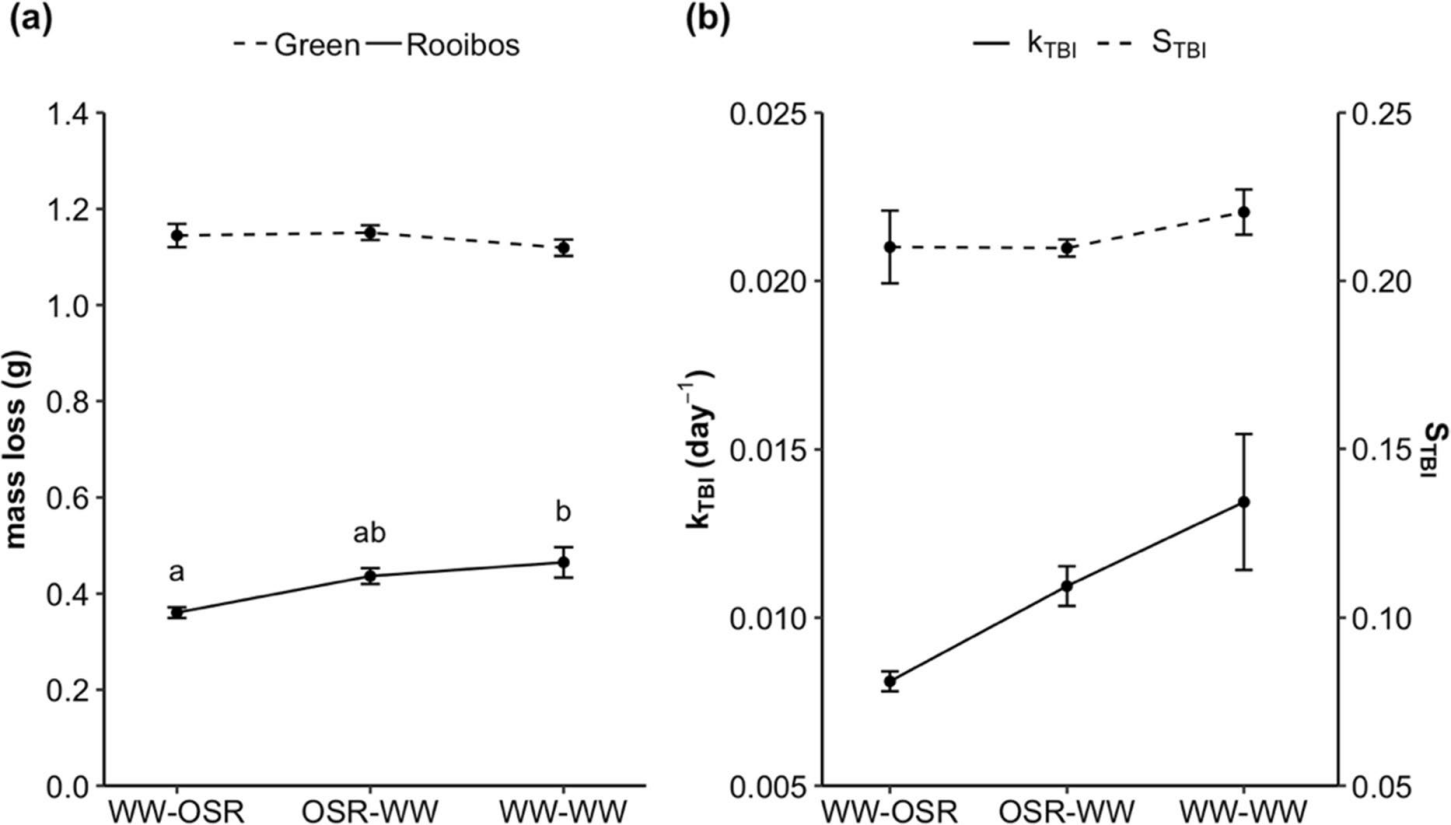
Mass loss of each tea type after 78 days in the different treatments (**a**); and the baseline decomposition rate constant (*k*_*TBI*_) and stabilisation factor (S_TBI_), as determined by the Tea Bag Index (TBI) protocol, for the different treatments (**b**). Error bars represent SEM (n = 4). Different letters indicate significant differences (post-hoc Tukey HSD, p < 0.05)

As expected from the principles that underlie the TBI protocol (Keuskamp et al. 2013), Green tea underwent more decomposition during the 78-day period than Rooibos tea (Fig. 7a), which can be attributed to the lower C:N ratio and greater hydrolysable fraction of Green tea. Green tea mass loss, which was used to calculate the stabilisation factor (*S*_*TBI*_), did not differ significantly between treatments (F_2,6_ = 0.55, p = 0.60). Rooibos mass loss, which was used to calculate the baseline decomposition rate (k_TBI_) of the different treatments, differed significantly between treatments (F_2,6_ = 5.25, p = 0.048). The values of *k*_*TBI*_ differed somewhat between treatments (F_2,6_ = 4.20, p = 0.072), increasing in the order WW-OSR < OSR-WW < WW-WW, with the greatest contrast between the WW-OSR treatment and the WW-WW treatment (p = 0.062, Tukey HSD) (Fig. 7b). This decomposition pattern observed for tea is contrary to observations made on the decomposition of OSR and wheat residues, which decomposed fastest in the WW-OSR treatment. The values of *S*_*TBI*_ did not differ significantly between treatments (F_2,6_ = 0.66, p = 0.55) (Fig. 7b).

## Discussion

### Absence of home-field advantage (HFA) within a short-rotation arable cropping system

We tested the HFA hypothesis between different stages of an arable cropping system using OSR and wheat residues in a reciprocal transplant experiment in a short crop rotation. According to the HFA hypothesis, which postulates a faster decomposition rate of *home* residues compared to residues that come from *away*, we expected OSR straw to decompose fastest in plots previously cropped with OSR (i.e. OSR-WW; see Table 1), and wheat straw to decompose fastest in plots previously cropped with wheat (i.e. WW-OSR and WW-WW; see Table 1). However, we observed no such differences and found that both residues decomposed similarly in all treatments (Fig. 3). Differences in the decomposition rates (*k*) between the treatments were not statistically significant and, more importantly, interactions between residue type and treatment that would indicate existence of an HFA were not statistically significant (p > 0.05), even after thorough investigation of the data (see Online Resource section S2). Therefore, we do not accept the HFA hypothesis in this experiment.

Greater chemical inputs (e.g. fertilisers and pesticides) in arable farming have allowed a move towards shorter and simpler crop rotations during the twentieth century (Robinson and Sutherland 2002; Tisdale et al. 1985). These intensively managed systems differ notably from more established uninterrupted vegetation assemblages in forests and permanent grasslands, where HFA effects have previously been observed (Ayres et al. 2009; Rashid et al. 2013). Arable cropping systems differ from grasslands and forests in the following ways: (1) they are frequently tilled, disturbing the soil structure and therefore disturbing the habitats of soil faunal species; (2) plant species are established for only one growing season and tend to be grown in a monoculture; (3) removal of grain and often crop residues at harvest reduces the amount of plant litter returned to the soil compared to natural systems; (4) soils are often left fallow for a period of time, as opposed to more continuous maintenance of vegetation in forests and grasslands so that (5) communities in arable soils are less well supplied with substrate, are likely to be patchy, less abundant, and poorly connected to their neighbours compared with communities in forests or grasslands (Simard et al. 1997); and (6) application of exogenous fertilisers, pesticides and fungicides impacts the soil microbial community. It has been suggested that an HFA may not be detected in soils that have been recently disturbed or where succession is relatively young (Austin et al. 2014; Gießelmann et al. 2011). Austin et al. (2014) pointed out that HFA effects are mostly observed in ecosystems without human disturbance, which is not the case in intensively managed arable cropping systems that are subject to ploughing, chemical inputs of fertilisers and pesticides, periods of fallow following crop harvest and, perhaps most importantly, have short periods of plant cover compared to perennial systems. All these activities impact on the soil microbial community.

Gießelmann et al. (2011) tested the HFA in forests at three successional stages with different tree species compositions and attributed the absence of an HFA to a soil microbial community that rapidly adapts to changes in litter quality additions. However, Veen et al. (2018), who also studied HFA at different successional stages, showed that the influence of successional stage on HFA depends on the conditions in the system considered, with high C:nutrient ratios in the litter and high SOM:nutrient ratios in the soil leading to greater HFA effects. Other studies identified contrasting litters and contrasting soil legacies as determinants of HFA effects (Li et al. 2017; Veen et al. 2015). Therefore, the absence of an HFA effect observed in our study could also be due to the similarity between the residue types and between the soil treatments.

### Residue quality and nutrient availability

Although no HFA was observed within the crop rotation in this experiment, we did observe a faster decomposition of wheat straw compared to OSR residues. This might be related to a legacy effect across the whole field site – rather than a plot-level soil microbial specialisation – which has a crop history in which wheat crops, and their residues, predominate. The faster decomposition of wheat straw can also be attributed to differences in the chemical characteristics of the two residues. Wheat straw exhibited a lower C:N ratio (Table 2), so microbial substrate use efficiency would have been higher (Cotrufo et al., 2013), because less soil-derived N would have been necessary to decompose the wheat residues compared to the OSR residues. Considering the C:N ratio of croplands and their microbial biomass have been estimated at 12.2 and 9.0, respectively (Xu et al. 2013), the C:N ratio of both residues in this study remains high. Although the average stoichiometric C:N requirements of soil microorganisms depends on the extent to which the microbial community is (able to) adapt to their substrate C:N ratio, soil microbial communities tend to become C limited when substrate C:N drops below about 17 and N limited when substrate C:N exceeds about 71 (Mooshammer et al. 2014). If the chemical quality of the residues was contrasting to the extent that one induced C limitation and the other induced N limitation, observing an HFA effect may have been more likely.

### Soil microbial community structure

In this study, the soil microbial community structure, determined by PLFA analysis, was similar in all treatments and changed similarly over time regardless of the crop rotation stage (Fig. 6), contrary to our second hypothesis. Ordination plots of the PLFA data did not identify the formation of a specific *home* microbial community that could be distinguished from the other soil treatments, nor did determinations of the total PLFAs, F:B ratio or G + :G– ratios. The lack of an identifiably different microbial community further supports the finding that an HFA effect could not be found in this system because the soil microbial community in each treatment was similar.

We did observe a shift over time in the microbial community structure that was similar for all treatments, suggesting an adaptation to environmental factors that occurred equally across all plots, such as changes in soil temperature, soil moisture, soil fertilisation status and crop development from the start of the experiment in February to the end of the experiment in July. A shift over time was also observed in the ratio of G + :G– bacteria. Fanin et al. (2019) found that the G + :G– ratio increased when sources of labile C were removed and they suggested a relationship between G– bacteria and simple organic substrates, and between G + bacteria and more complex organic substrates; thus, the ratio of G + :G– bacteria may indicate lability of organic carbon. In our experiment, the amount of both G + and G– bacterial fatty acids increased over time, but the increase was greater for G + bacterial fatty acids, suggesting the soil microbial community may have adapted to being able to decompose more complex forms of C during the period the mesh bags were in the soil. This observation can most likely be attributed to the progression of root detritus and stubble decomposition in the soils. Easily decomposable substrates were probably metabolised first, such that the more complex organic compounds were left towards the end of the experimental period.

Adaptation of the soil microbial community, to which the existence of an HFA is attributed, is typically considered to be a long-term process because specialisation may decrease the functional redundancy of a community (Keiser et al. 2011), and therefore decrease the ability of the community to adapt to environmental changes (like inputs of organic substrates with different chemical qualities). There tends to be a link between habitat specialisation and life strategies, with *r*-strategists being generalists and *K*-strategists being specialists (McKinney 1997; Sakai et al. 2001). It has also been suggested that even low residual abundances of species adapted to the decomposition of the residues of crops that grew in the soil several seasons earlier can regenerate relatively quickly in response to a new litter input (Gießelmann et al. 2011). Therefore, it may be that the soil microbial community in the arable cropping system in this experiment is composed of mostly *r*-selected species that are not specialised for a particular environment (Blagodatskaya and Kuzyakov 2008; Bowers and Harris 1994). This proposition, if true, can be attributed to soil disturbance and changes in residue inputs from year to year that have prevented the establishment of a soil microbial community including specialised *K*-selected species within the soil microbial community.

Fungi represent one group of microbes that are generally thought of as *K*-strategists (Fontaine et al. 2003), although this is not always the case (Fierer et al. 2007). Lin et al. (2018) found fungi to play a prominent role in driving HFA effects for not only low-quality but also high-quality litters in broadleaf and bamboo forests. This finding could be due to the fungi being *K*-selected and significantly contributing to specialisation of the soil microbial community. There were no significant differences in fungal biomarkers in the soil treatments in our study (also see Online Resource section S4). Inorganic fertilisers have been shown to lead to decreased fungal- and increased bacterial richness (Chen et al. 2016), and higher F:B ratios are generally observed in less disturbed systems (Gregory et al. 2009). Therefore, the role of fungi and/or *K*-strategists in general, might be supressed in intensively managed arable cropping systems, eliminating HFA legacy effects.

### Tea Bag Index (TBI)

The TBI can be used as a comparative measure of soil microbial activity between contrasting sites, where *k*_*TBI*_ is representative of the ability of the soil decomposer community and *S*_*TBI*_ is an indication of the degree of inhibition from environmental conditions on the decomposition of labile substrates (Keuskamp et al. 2013). The *k*_*TBI*_ and *S*_*TBI*_ values reported in this experiment are within the range of values expected from the protocol (Keuskamp et al., 2013). There were no significant differences in *k*_*TBI*_ between the different treatments, suggesting the microbial ability to decompose was similar in all treatments (Fig. 7b). However, the mass loss of Rooibos tea was lower in the WW-OSR treatment, compared to the OSR-WW treatment, and significantly lower compared to the WW-WW treatment (Fig. 7a). Therefore, decomposition may have been inhibited in the WW-OSR treatment. As a brassica species, OSR releases isothiocyanates (biofumigant glucosilonate compounds) upon decomposition, so the decomposing OSR residue may have had a biofumigant effect and negatively influenced decomposer activity leading to a lower decomposition rate of the OSR residues themselves. However, residue decomposition in mesh bags containing OSR residues would not have affected the decomposition of tea buried separately in teabags, and therefore cannot explain the low *k*_*TBI*_ in WW-OSR.

### HFA in different arable crop rotations

Studies on the HFA hypothesis are highly variable in their results, with many reports on both the presence (Ayres et al. 2009; Lin et al. 2018) and absence (Ayres et al. 2006; Gießelmann et al. 2011; St. John et al. 2011) of an HFA, both within and between ecosystems. Therefore, despite not observing an HFA effect in this experiment, we do not reject the possibility of the mechanisms underpinning the HFA playing a role in crop residue decomposition in some arable cropping systems. Although we found no HFA between the different treatments, wheat straw exhibited a higher decomposition rate than OSR straw in all plots, which could be related to a historically greater exposure to wheat crops and residues in the field site (legacy effect). If that is the case, the HFA effect might still apply to arable cropping systems, but between different crop rotations composed of different crops, rather than between the different stages of a crop rotation.

The HFA hypothesis could be further tested in a future experiment where (1) both residue types are buried in a crop rotation dominated by wheat as well as in a different crop rotation dominated by another crop; or (2) within a rotational system that includes crops with more contrasting residue chemistries; or (3) in a perennial system where plants are established for longer periods of time. Where and how an HFA applies depends on the mechanisms that underlie the specialisation of the microbial community responsible for the HFA effect, which remain poorly understood (Austin et al. 2014). Proposed mechanisms include litter-decomposer interactions ranging from (1) microbial selection via root exudates or plant litter volatiles, (2) “green-leaf hitchhikers” that persist from green leaves/stalks to the litter stage, and (3) three-way interactions where plants influence soil microbial and microarthropod communities, whose frass (arthropod excrement) production further selects for microbes (Austin et al. 2014). Further experiments in different arable cropping systems and alternative research methods (e.g. field-scale amendment with residues instead of litterbags) are needed to determine in what circumstances (if any) an HFA can be observed within an arable cropping system.

## Conclusions

The decomposition rates of wheat and OSR residues in a reciprocal transplant experiment within a short-rotation arable cropping system did not reveal an HFA effect. We mainly attribute this to the similarity of the soil microbial community structures between the different treatments (i.e. different stages of a short arable crop rotation). The lack of an HFA effect could be due to high levels of soil disturbance and short duration of crop growth in these intensively managed systems. We further suggest that the decomposition rates observed here may be better explained by the differences in the chemical quality (e.g. C:N ratio) of wheat residues compared to OSR residues, and the levels of soil available N in each treatment.

Therefore, this study provides no evidence that rotating crop residues out of sync with the crop rotation can realise an “away-field disadvantage” with greater net SOM accumulation in a short conventionally managed arable rotation.

## Supplementary Information

Below is the link to the electronic supplementary material.

Supplementary file1 (DOCX 3515 kb)

## Data Availability

Mendeley Data https://doi.org/10.17632/3md3548679

## Code availability

n/a.
