# Supplementary material for: Absence of a home-field advantage within a short-rotation arable cropping system
Source: Plant Soil. 2022 Apr 26;488(1-2):39–55. doi: 10.1007/s11104-022-05419-z (PMC10435649; doi:10.1007/s11104-022-05419-z)
Supplement: Supplementary file 1 — Supplementary file1 (DOCX 3515 kb) [file 11104_2022_5419_MOESM1_ESM.docx]

Supplement to Absence of a home-field advantage within a short‑rotation arable cropping system

**Marijke Struijk^1,2^, Andrew P. Whitmore^2^, Simon Mortimer^3^, Xin Shu^1^ and Tom Sizmur^1^**

^1^ Department of Geography and Environmental Science, University of Reading, Reading, UK.

^2^ Department of Sustainable Agriculture Sciences, Rothamsted Research, Harpenden, UK.

^3^ School of Agriculture, Policy and Development, University of Reading, Reading, UK.

*Corresponding author*: Marijke Struijk (marijkestruijk@gmail.com)

Table of contents

**S1.** Schedule of the experiment 2

**S2.** Statistical analyses for testing HFA hypothesis 3

**S3.** Alternative treatment designation 6

**S4.** Additional analyses of PLFA data 7

**S5.** SAS output from model proposed by Keiser et al. (2014) for analysis of HFA effects 9

S1. Schedule of the experiment

| **Table S5.** Schedule of the experiment with the months and days referred to in text and figures. | | | |
| --- | --- | --- | --- |
| **Event** | **Sampling month** | **Experimental day** | **Date** |
| Planting of OSR |  | n/a | 11 September 2015 |
| Planting of wheat |  | n/a | 16 October 2015 |
| Mesh bags buried (blocks 1, 2, 4)  Mesh bags buried (block 3) |  | 0  2 | 10 February 2016  12 February 2016 |
| Initial soil samples (blocks 3 & 4)  Initial soil samples (blocks 1 & 2) | Initial  Initial | 2  5 | 12 February 2016  15 February 2016 |
| PLFA soil samples (start) |  | 23 | 4 March 2016 |
| Mesh bag retrieval 1 | 1 | 26 | 7 March 2016 |
| Mesh bag retrieval 2 | 2 | 54 | 4 April 2016 |
| Fertilisation of wheat plots |  | 58 | 8 April 2016 |
| Fertilisation of OSR plots |  | 70 | 20 April 2016 |
| Mesh bag retrieval 3 | 3 | 82 | 2 May 2016 |
| Teabags buried |  | 102 | 22 May 2016 |
| Mesh bag retrieval 4 | 4 | 110 | 30 May 2016 |
| Mesh bag retrieval 5 | 5 | 138 | 27 June 2016 |
| PLFA soil samples (end) |  | 146 | 5 July 2016 |
| Mesh bag retrieval 6 | 6 | 166 | 25 July 2016 |
| Teabags retrieved |  | 180 | 8 August 2016 |

S2. Statistical analyses for testing HFA hypothesis

| **Table S1.** Results of one-way ANOVAs attempted to test HFA hypothesis. Assumptions of normal distribution of residuals and equal variance tested with Shapiro-Wilk and Levene tests, respectively. Blocking structure is included as error factor. Other factors included in the model are specified. Significance indicated as **p < 0.05** and p < 0.1. | | | | |
| --- | --- | --- | --- | --- |
|  | **Y = wheat *k*** | | **Y = OSR *k*** | |
| **Factors** | **F** | **p** | **F** | **p** |
| Treatment | 1.191 | 0.353 | 0.524 | 0.611 |
| Treatment  Covariate: average available N | 1.155  0.760 | 0.369  0.412 | 0.964  7.710 | 0.427  **0.027** |
| Treatment  Covariate: k_TBI_ | 1.060  0.124 | 0.396  0.735 | 0.493  0.515 | 0.631  0.496 |
| Treatment  Covariate: S_TBI_ | 1.050  0.055 | 0.399  0.822 | 0.473  0.214 | 0.642  0.657 |
| Treatment  Covariate: PLFA biomass start | 1.042  0.001 | 0.402  0.971 | 0.480  0.317 | 0.638  0.591 |
| Treatment  Covariate: PLFA biomass end | 1.603  3.769 | 0.267  0.093 | 0.472  0.207 | 0.642  0.663 |
| Treatment  Covariate: F:B start | 0.989  0.089 | 0.435  0.777 | 0.516  0.027 | 0.625  0.876 |
| Treatment  Covariate: F:B end | 0.973  0.004 | 0.440  0.951 | 0.651  1.337 | 0.561  0.300 |
| Treatment  Covariate: C18:1ω9c start | 0.987  0.076 | 0.435  0.793 | 0.549  0.343 | 0.609  0.584 |
| Treatment  Covariate: C18:1ω9c end | 1.339  1.891 | 0.342  0.228 | 0.646  1.286 | 0.563  0.308 |
| Treatment  Covariate: C18:2ω6c start | 1.069  0.499 | 0.411  0.511 | 0.515  0.009 | 0.626  0.929 |
| Treatment  Covariate: C18:2ω6c end | 1.064  0.474 | 0.412  0.522 | 0.742  2.223 | 0.522  0.196 |

Table S1 details the results of the range of analyses of variance (ANOVAs) performed to determine if any home-field advantage (HFA) effect could be detected. A range of covariates that could reasonably affect decomposition or be representative of the decomposition ability of the soil were also tested to find out if this would improve prediction of the decomposition rate constant by the treatment (treatment).

| **Table S2.** Results of two-way ANOVAs attempted to test HFA hypothesis. Assumptions of normal distribution of residuals and equal variance tested with Shapiro-Wilk test and Levene test, respectively. Y‑variate is *k*. Blocking structure is included as error factor. Other factors included in the model are specified. Significance indicated as **p < 0.05** and p < 0.1. | | |
| --- | --- | --- |
| **Factors** | **F** | **p** |
| Treatment  Residue  Treatment × residue | 1.582  18.738  0.194 | 0.238  **<0.001**  0.825 |
| Treatment  Residue  Covariate: average available N  Treatment × residue | 2.337  27.674  8.153  0.287 | 0.133  **<0.001**  **0.013**  0.755 |
| Treatment  Residue  Covariate: k_TBI_  Treatment × residue | 1.526  18.072  0.466  0.187 | 0.251  **<0.001**  0.506  0.931 |
| Treatment  Residue  Covariate: S_TBI_  Treatment × residue | 1.479  17.513  0.019  0.182 | 0.261  **<0.001**  0.893  0.836 |
| Treatment | 1.816 | 0.195 |
| Residue | 21.510 | **<0.001** |
| Covariate: Rooibos mass loss | 1.314 | 0.268 |
| Treatment × residue | 0.223 | 0.803 |
| Treatment | 1.699 | 0.214 |
| Residue | 20.119 | **<0.001** |
| Covariate: Rooibos mass loss | 0.665 | 0.665 |
| Treatment × residue | 0.814 | 0.814 |
| Treatment  Residue  Covariate: PLFA biomass start  Treatment × residue | 1.497  17.732  0.194  0.184 | 0.257  **<0.001**  0.666  0.834 |
| Treatment  Residue  Covariate: PLFA biomass end  Treatment × residue | 1.891  22.393  3.925  0.232 | 0.188  **<0.001**  0.068  0.796 |
| Treatment  Residue  Covariate: Increase in PLFA biomass  Treatment × residue | 1.513  17.917  0.342  0.186 | 0.254  **<0.001**  0.568  0.832 |

| **Table S2.** continued | | |
| --- | --- | --- |
| Treatment  Residue  Covariate: F:B start  Treatment × residue | 1.479  17.515  0.021  0.182 | 0.261  **<0.001**  0.887  0.836 |
| Treatment  Residue  Covariate: F:B end  Treatment × residue | 1.543  18.270  0.625  0.189 | 0.248  **<0.001**  0.442  0.829 |
| Treatment  Residue  Covariate: G+:G– start  Treatment × residue | 1.542  18.259  0.617  0.189 | 0.248  **<0.001**  0.445  0.830 |
| Treatment  Residue  Covariate: G+:G– end  Treatment × residue | 1.485  17.587  0.078  0.182 | 0.260  **<0.001**  0.783  0.835 |

To assess presence of a HFA effect, a range of ANOVAs was performed. Neither of the residues were significantly affected by treatment (OSR: F = 0.62, p = 0.57; wheat: F = 1.17, p = 0.37; one-way ANOVA of each residue). For a HFA effect to be present, a significant interaction between the treatment and residue type in a two-way ANOVA is necessary, so this test was chosen as the initial method of detection. Decomposition rates of wheat were significantly higher than oilseed rape (OSR) (F = 18.74, p < 0.001). However, the treatment had no significant effect on decomposition (F = 1.58, p = 0.24; two-way ANOVA) and neither did the interaction of *treatment* and *residue*.

Because we expected the fungal:bacterial (F:B) ratio of the soil to be related to the decomposition rate, a two-way ANOVA of *k* and treatment with F:B ratio as a covariate was performed, but this led to no significant results. Because soil available N plays a major factor in the ability of the soil microbial community to decompose residues, the average available N during the experimental period was calculated and taken into account as a covariate of *k*. However, this did not affect the significance of the interactive effect of treatment × residue.

S3. Alternative treatment designation

It is unlikely but possible that the treatment designation for wheat straw follows a different logic than that of OSR straw, where the home field of OSR residue decomposition is a soil with OSR crop, while the home field of wheat straw is a soil with a wheat crop in the previous year. If this is true, and the treatment designation is adjusted, the results of a two-way ANOVA with and without available N as a covariate are summarised in Table S3.

| **Table S3.** Results of two-way ANOVAs if treatment designation is different for decomposing wheat straw. Y-variate is *k*. Blocking structure is included as error factor. Other factors included in the model are specified. Significance indicated as **p < 0.05** and p < 0.1. | | |
| --- | --- | --- |
| **Factors** | **F** | **p** |
| Treatment  Residue  Treatment × residue | 0.437  18.738  1.339 | 0.654  **<0.001**  0.292 |
| Treatment  Residue  Covariate: average available N  Treatment × residue | 0.664  27.674  4.866  3.621 | 0.539  **<0.001**  **0.045**  0.054 |
| Treatment  Residue  Covariate: k_TBI_  Treatment × residue | 0.422  18.072  2.626  0.212 | 0.664  **<0.001**  0.127  0.812 |
| Treatment  Residue  Covariate: PLFA biomass end  Treatment × residue | 0.522  22.393  0.896  3.115 | 0.604  **<0.001**  0.360  0.076 |

From these results the presence of an HFA effect is possible with a 90% confidence interval if designation of the treatments in a crop rotation is different for different residues.

This is the only method of data analysis that has yielded an almost significant interactive effect of treatment × residue. However, to assign different treatments to the wheat residue is an artificial, speculative and highly doubtful approach.

S4. Additional analyses of PLFA data

| **Table S4.** Fatty acids that were significantly or noticeably affected by the factors Time and/or Treatment. Significance indicated as **p < 0.05** and p < 0.1. Biomarker information taken from Bååth and Anderson, 2003; Frostegård and Bååth, 1996; Frostegard *et al*., 1993; Bardgett *et al*., 1999. | | | | |
| --- | --- | --- | --- | --- |
| **Biomarker** | **Fatty acid** | **F** | **p** | **Factor** |
|  | C14:1ω9c | 84.37 | **<0.001** | Time |
| Bacteria, G+ | C15:0i | 21.74 | **<0.001** | Time |
| Bacteria, G+ | C15:0ai | 26.63  4.52 | **<0.001**  **0.030** | Time  Treatment |
| Bacteria | C15:0 | 20.67 | **<0.001** | Time |
| Bacteria, G+ | C16:0i | 35.89  2.80 | **<0.001**  0.088 | Time  Treatment |
| G– | C16:1ω7c | 7.30 | **0.015** | Time |
| Bacteria, G– | C16:1ω7t | 31.22 | **<0.001** | Time |
| AMF and bacteria, G+ | C16:1ω5 | 9.97 | **0.005** | Time |
|  | C16:0 | 41.00  2.82 | **<0.001**  0.086 | Time  Treatment |
|  | C17:0brα | 7.42 | **0.014** | Time |
| Bacteria, G+ | C17:0i | 40.72 | **<0.001** | Time |
|  | C17:0brβ | 27.74  2.69 | **<0.001**  0.095 | Time  Treatment |
|  | C17:1ω8c | 6.86 | **0.017** | Time |
| Bacteria, G– | C17:0cy | 34.92 | **<0.001** | Time |
| G– | C17:1ω7 | 9.41  3.14 | **0.007**  0.067 | Time  Treatment |
| Bacteria | C17:0 | 54.14 | **<0.001** | Time |
|  | C17:0-12me | 11.45 | **0.003** | Time |
| Actinomycetes, G+ | C17:0-10me | 51.86 | **<0.001** | Time |
|  | C18:3-5,10,12 | 15.79 | **<0.001** | Time |
| Fungi | C18:1ω9c | 7.18 | **0.020** | Time |
|  | C18:1ω9t | 3.50 | 0.080 | Time |
|  | C18:1ω13 | 20.62  4.16 | **<0.001**  **0.033** | Time  Treatment |
|  | C18:1ω10or11 | 3.81 | 0.067 | Time |
|  | C18:0 | 48.67 | **<0.001** | Time |
|  | C19:1ω6 | 3.72 | 0.070 | Time |
| Actinomycetes, G+ | C18:0-10me | 51.88 | **<0.001** | Time |
|  | C19:1ω8 | 38.56 | **<0.001** | Time |
|  | C19:0 | 9.43 | **0.007** | Time |
| Bacteria | C19:0cy | 2.95 | 0.078 | Treatment |
| Protists | C20:4ω6 | 12.72 | **0.002** | Time |
|  | C20:5ω3 | 15.86 | **<0.001** | Time |
|  | C20:1ω9 | 13.07 | **0.002** | Time |
|  | C20:0 | 31.89  3.33 | **<0.001**  0.059 | Time  Treatment |
|  | Total of all FAs | 17.93 | **<0.001** | Time |

| 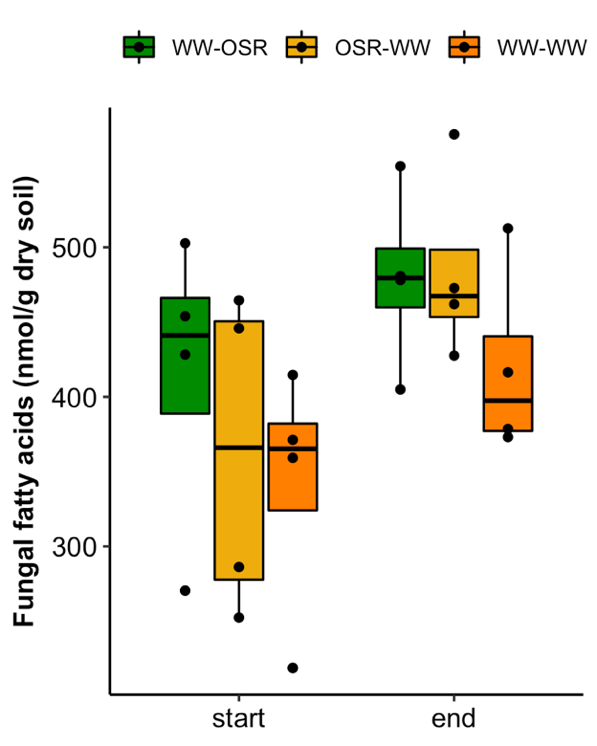 | 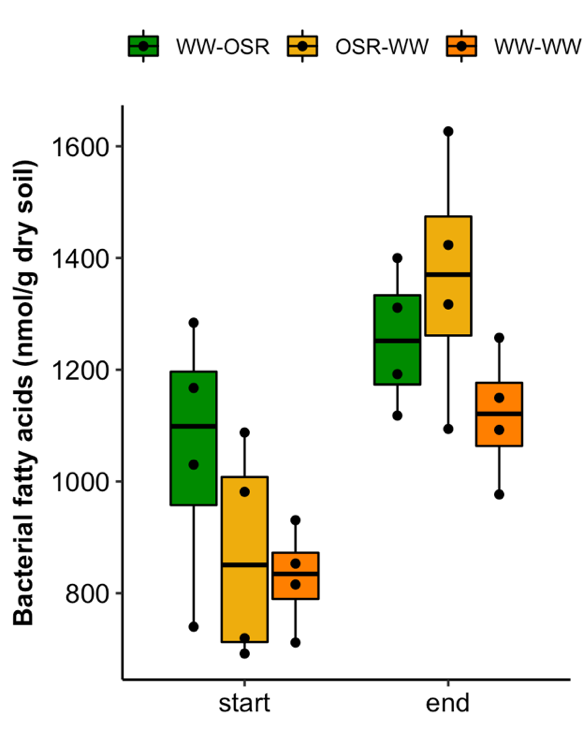 |
| --- | --- |
| **Figure S1.** Biomass of fungal PLFAs per treatment at the start (March 2016) and end (July 2016) of the growing season. Lower and upper hinges correspond to the 25th and 75th percentiles; black dots represent individual datapoints (n = 4). | **Figure S2.** Biomass of bacterial PLFAs per treatment at the start (March 2016) and end (July 2016) of the growing season. Lower and upper hinges correspond to the 25th and 75th percentiles; black dots represent individual datapoints (n = 4). |

There was a significant difference between the start and the end of the experimental period for both fungal fatty acids (F = 7.520; p = 0.126) and bacterial fatty acids (F = 20.551, p < 0.001; two-way ANOVA). There were no significant differences between treatments. There was a good correlation between the fungal biomarkers 18:2ω6 and 18:1ω9.

| 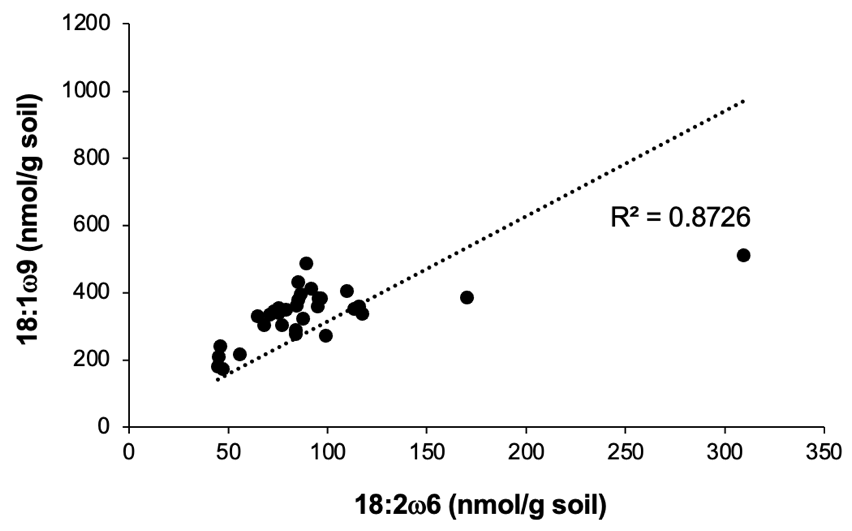 |
| --- |
| **Figure S3.** Correlation between fungal biomarkers. |

S5. SAS output from model proposed by Keiser et al. (2014) for analysis of HFA effects

| **Number of Observations Read** | 288 |
| --- | --- |
| **Number of Observations Used** | 288 |

| **Analysis of Variance** | | | | | |
| --- | --- | --- | --- | --- | --- |
| **Source** | **DF** | **Sum of Squares** | **Mean Square** | **F Value** | **Pr > F** |
| **Model** | 5 | 0.08858 | 0.01772 | 0.50 | 0.7787 |
| **Error** | 282 | 10.06116 | 0.03568 |  |  |
| **Corrected Total** | 287 | 10.14974 |  |  |  |

| **Root MSE** | 0.18889 | **R-Square** | 0.0087 |
| --- | --- | --- | --- |
| **Dependent Mean** | 0.55853 | **Adj R-Sq** | -0.0088 |
| **Coeff Var** | 33.81854 |  |  |

| **Parameter Estimates** | | | | | | | | |
| --- | --- | --- | --- | --- | --- | --- | --- | --- |
| **Variable** | **DF** | **Parameter Estimate** | **Standard Error** | **t Value** | **Pr > \|t\|** | **Heteroscedasticity Consistent** | | |
|  |  |  |  |  |  | **Standard Error** | **t Value** | **Pr > \|t\|** |
| **Intercept** | **B** | 0.55633 | 0.01928 | 28.86 | <.0001 | 0.01911 | 29.12 | <.0001 |
| **SoilW1** | **B** | 0.00084466 | 0.02226 | 0.04 | 0.9698 | 0.02226 | 0.04 | 0.9698 |
| **SoilW2** | **B** | 0.00336 | 0.02226 | 0.15 | 0.8801 | 0.02138 | 0.16 | 0.8752 |
| **SoilO** | **B** | -0.00421 | 0.02226 | -0.19 | 0.8503 | 0.02223 | -0.19 | 0.8501 |
| **LitterW1** | **B** | -0.01885 | 0.01928 | -0.98 | 0.3291 | 0.01941 | -0.97 | 0.3325 |
| **LitterW2** | **0** | 0 | . | . | . | . | . | . |
| **LitterO** | **1** | 0.01885 | 0.01928 | 0.98 | 0.3291 | 0.01941 | 0.97 | 0.3325 |
| **HFAW1** | **1** | 0.00328 | 0.05453 | 0.06 | 0.9520 | 0.05421 | 0.06 | 0.9517 |
| **HFAW2** | **1** | 0.00990 | 0.05453 | 0.18 | 0.8561 | 0.05418 | 0.18 | 0.8551 |
| **HFAO** | **0** | 0 | . | . | . | . | . | . |
| **RESTRICT** | **-1** | -3.7924E-14 | 4.875062E-9 | -0.00 | 1.0000* | . | . | . |
| **RESTRICT** | **-1** | -7.816E-14 | 0 | . | . | . | . | . |

| *** Probability computed using beta distribution.** |
| --- |
